# Management of important adverse events associated with inotuzumab ozogamicin: expert panel review

**DOI:** 10.1038/s41409-017-0019-y

**Published:** 2018-01-12

**Authors:** Partow Kebriaei, Corey Cutler, Marcos de Lima, Sergio Giralt, Stephanie J. Lee, David Marks, Akil Merchant, Wendy Stock, Koen van Besien, Matthias Stelljes

**Affiliations:** 1 University of Texas MD Anderson Cancer Center, Houston, TX USA; 2 Dana Farber Cancer Center, Boston, MA USA; 3 UH Cleveland Medical Center and Case Western Reserve University, Cleveland, OH USA; 4 Memorial Sloan Kettering, New York, NY USA; 5 Fred Hutchinson Cancer Research Center, Seattle, WA USA; 6 University Hospitals Bristol, Bristol, UK; 7 University of Southern California, Los Angeles, CA USA; 8 University of Chicago, Chicago, IL USA; 9 Weill Cornell Medical College, New York, NY USA; 10 Universitätsklinikum Münster, Münster, Germany

## Abstract

Inotuzumab ozogamicin (InO), a humanized anti-CD22 monoclonal antibody conjugated to the cytotoxic antibiotic agent calicheamicin, has demonstrated efficacy in the phase 3 INO-VATE study of adults with relapsed or refractory acute lymphoblastic leukemia (ALL). Findings from the study showed clinically important adverse events (AEs) associated with InO, with veno-occlusive disease (VOD) reported as a major non-hematologic AE. Other important or serious AEs include neutropenia, febrile neutropenia, thrombocytopenia, infusion-related reactions, tumor lysis syndrome, and prolonged QT syndrome. This report summarizes the recommendations of an expert panel of hematologists and transplant physicians for evaluation and management of the important AEs associated with InO, with a focus on diagnosis, prevention, monitoring, and management of VOD. The possible interventions considered included prophylaxis medications, patient monitoring and assessment, and InO dose adjustment or discontinuation.

## Introduction

Inotuzumab ozogamicin (InO) is composed of an anti-CD22 monoclonal antibody conjugated to calicheamicin, a highly potent cytotoxic antibiotic [1]. InO was developed and studied for its potential benefits in patients with B-cell acute lymphoblastic leukemia (ALL). Findings from INO-VATE, a phase 3, open-label, randomized study, showed the overall tolerability and superior efficacy of InO compared with the investigator’s choice of standard chemotherapy as first or second salvage treatment in adults with relapsed or refractory, Philadelphia chromosome (Ph)‒positive or Ph-negative B-cell ALL [2]. However, several clinically important adverse events (AEs) or serious AEs associated with InO were reported (Supplementary Table 1) [2, 3]. Most importantly, sinusoidal obstruction syndrome, or veno-occlusive disease (VOD), occurring during and after treatment, with or without subsequent hematopoietic cell transplantation (HCT), was more common in the InO arm compared with standard therapy (13 vs <1%) [4].

Veno-occlusive disease associated with InO is especially a concern among patients who proceed to HCT [2]. Findings from the INO-VATE study showed that 77 of the 164 patients (47%) in the InO arm proceeded to HCT compared with 33 of 162 patients (20%) in the standard therapy arm (*P* < 0.0001) [4]. Of these 77 patients in the InO arm, 17 (22%) developed VOD, with 5 fatal cases [4]. The primary goal in patients with relapsed ALL is to produce a durable complete remission with or without minimal residual disease to enable patients to proceed to more curative treatment, such as HCT [5]. It is therefore important for all physicians who administer InO to be able to identify and manage VOD and, if possible, to prevent it, especially in patients who have the goal of proceeding to HCT.

To reach a consensus on guidelines in the prevention, evaluation, and management of VOD and other important AEs associated with InO, a meeting of expert oncologists and hematologists, including transplant specialists, was held in Orlando, Florida, on 21 February 2017. This article describes the recommendations of the expert panel, with an emphasis on the diagnosis and management of VOD in patients treated with InO. In addition, this report summarizes the incidences of important AEs associated with InO based on a safety population of 259 patients (139 patients in the InO arm and 120 patients in the standard-therapy arm) from the INO-VATE study (data cutoff date: October 2, 2014) [2]. The incidence of VOD was based on findings from the INO-VATE study in an updated safety population of 307 patients (164 patients in the InO arm and 143 patients in the standard-therapy arm; data cutoff date: March 8, 2016) [4].

## Veno-occlusive disease

Veno-occlusive disease, also known as sinusoidal obstruction syndrome, is a serious and potentially fatal liver-related complication of HCT, with a previous analysis reporting an 84% mortality rate from severe VOD [6]. In the INO-VATE study, the incidence of any grade and grade ≥3 VOD (based on the National Cancer Institute Common Terminology Criteria for Adverse Events, version 3.0) was higher in the InO group (13 and 11%, respectively) compared with the standard-therapy group (<1 and <1%, respectively) [4]. In the InO group, the median time to VOD onset after the first InO dose was 30 days (range: 14–238 days) [3]. The median onset of VOD was 15 days after HCT among patients in the safety population receiving InO who proceeded to follow-up HCT [4]. VOD after follow-up HCT was reported in 8% of patients receiving 1 InO cycle, 19% of patients receiving 2 cycles of InO, and 29% of patients receiving >2 InO cycles [4].

VOD can result from damage to the sinusoidal endothelium and hepatocytes due to toxic metabolites generated by high-dose alkylating chemotherapy conditioning regimens [7]; however, the pathophysiology of VOD associated with InO is not completely understood. It has been shown that gemtuzumab ozogamicin, a humanized anti-CD33 antibody, also conjugated to calicheamicin, is associated with VOD [8]. VOD associated with InO and gemtuzumab ozogamicin may potentially be due to a direct effect of calicheamicin on sinusoidal endothelial cells [8].

Weight gain from renal retention of water and sodium is one of the earliest signs of VOD [9]. Additional signs and symptoms of VOD include jaundice, painful hepatomegaly, edema, and ascites [10, 11]. Other less common findings suggestive of typically severe VOD include hypoxia, encephalopathy, pleural effusion, pulmonary infiltrates, thrombocytopenia, and renal insufficiency or failure, which can eventually result in multi-organ failure [9, 10]. The onset of VOD is defined as classic if VOD occurs within 21 days after HCT and late onset if it occurs >21 days after HCT [12]. On the basis of the new European Society for Blood and Marrow Transplantation (EBMT) guidelines, the diagnostic criteria for VOD depend on clinical and laboratory findings and whether VOD is classical or late onset (Table 1) [12]. The severity of VOD is determined by bilirubin level and its rate of change, liver function, weight gain, and renal function as well as the kinetics of VOD onset (Table 1) [12].

**Table 1:** Criteria for VOD diagnosis and grading [12]

EBMT Criteria for Diagnosing VOD	
Classical VOD	Late-onset VOD
In the first 21 days after HCT	>21 days after HCT
Bilirubin ≥2 mg/dL and 2 of the following criteria^a^:	Classical VOD beyond d 21 or
Painful hepatomegaly	Histologically proven VOD
Weight gain >5%	or
Ascites	≥2 of the following criteria^a^:
	Bilirubin ≥2 mg/dL (or 34 μmol/L)
	Painful hepatomegaly
	Weight gain >5%
	Ascites
	and
	Hemodynamic and/or ultrasound evidence of VOD

Grading of VOD Severity				
Endpoints	VOD Grade			
	Mild^b^	Moderate^b^	Severe^b^	Very Severe^c^
Time since first clinical symptoms of VOD^d^	>7 days	5‒7 days	≤4 days	Any time
Bilirubin, mg/dL	≥2 and <3	≥3 and <5	≥5 and <8	≥8
Bilirubin, μmol/L	≥34 and <51	≥51 and <85	≥85 and <136	≥136
Bilirubin kinetics	Doubling within 48 h			
Transaminases	≤2× normal	>2 and ≤5× normal	>5 and ≤8× normal	>8× normal
Weight increase	<5%	≥5% and <10%	≥5% and <10%	≥10%
Renal function	<1.2× baseline at transplant	≥1.2 and <1.5× baseline at transplant	≥1.5 and <2× baseline at transplant	≥2× baseline at transplant or other signs of MOD/MOF

Patients belong to the severity category that fulfills ≥2 criteria. If patients fulfill ≥2 criteria in 2 different categories, they must be classified in the most severe category. Patients’ weight increase ≥5 and<10% is considered by default as a criterion for severe VOD; however, if patients do not fulfill other criteria for severe VOD, weight increase ≥5 and <10% is therefore considered as a criterion for moderate VOD

*EBMT* European Society for Blood and Marrow Transplantation, *HCT* hematopoietic cell transplantation, *MOD* multi-organ dysfunction, *MOF* multi-organ failure, *VOD* veno-occlusive disease

^a^These signs or symptoms should not be attributable to other causes

^b^In the case of presence of ≥2 risk factors for VOD, patients should be in the upper grade

^c^Patients with MOD must be classified as very severe

^d^Time from the date when the first signs/symptoms of VOD began to appear (retrospectively determined) and the date when the symptoms fulfilled VOD diagnostic criteria

Established risk factors for VOD include use of myeloablative, busulfan-based (typically orally and non-pharmacokinetically dosed), or total body irradiation-based conditioning regimens; older age; Karnofsky score <90%; advanced disease (beyond second complete remission or relapse); and active viral hepatitis [10]. A recent study conducted by the Center for International Blood and Marrow Transplant Research (CIBMTR) developed and assessed a risk score to identify patients receiving allogeneic HCT at high risk for VOD [13]. On the basis of risk factors of age, hepatitis B/C serology, Karnofsky performance score, disease type/status, conditioning regimen, and sirolimus use, patients were classified into four groups based on their risk score (low, intermediate, high, or very-high risk for developing VOD). InO and other calicheamicin-based antibody-drug conjugates were not considered in this analysis because low numbers of patients were exposed to these treatments. The risk score was valid in successfully stratifying patients and identifying those at high risk of developing VOD.

## Recommendations

### Preventing veno-occlusive disease

As soon as it is decided that a patient will be initiating InO treatment, the patient should be referred for HCT evaluation. In patients for whom HCT is considered, the number of InO cycles should be limited to 2, if feasible, because the rate of VOD increases with increasing cycles of InO (Table 2) [4]. In addition, conditioning regimens containing dual alkylating agents (e.g., thiotepa and melphalan) should be avoided if possible, because the use of dual alkylator regimens was identified as a risk factor for developing VOD based on multivariate analysis findings in the INO-VATE study [4]. When possible, we also recommend avoiding hepatotoxic agents (eg, azoles) in combination with high-dose alkylator-conditioning administration. Finally, it is recommended that pharmacologic agents (e.g., ursodiol) be given to all patients exposed to InO to prevent VOD.

**Table 2:** Recommendations for preventing and monitoring VOD in patients receiving InO

Preventing VOD	
•	Avoid HCT conditioning regimens containing dual alkylating agents, thiotepa, or both
•	Use prophylactic agents (e.g., ursodiol)
•	When possible, avoid hepatotoxic agents (e.g., azoles) in combination with high-dose alkylator-condition administration
•	In patients proceeding to HCT, limit treatment with InO to 2 cycles
Monitoring for VOD	
•	In patients who have experienced prior confirmed severe or ongoing VOD, follow recommendations in country-specific prescribing information to determine appropriate use of InO
•	Monitor patient weight for fluid retention daily
•	More frequently monitor LFTs and look for clinical signs and symptoms of hepatotoxicity in patients who develop abnormal LFTs
•	Before and after each InO dose, monitor ALT, AST, total bilirubin, and alkaline phosphatase levels and adjust InO dose as recommended (Table 3)
•	In patients proceeding to HCT, closely monitor LFTs during the first month post-HCT, then less frequently thereafter based on standard practice

*ALT* alanine aminotransferase, *AST* aspartate aminotransferase, *HCT* hematopoietic cell transplantation, *InO* inotuzumab ozogamicin, *LFT* liver function test, *ULN* upper limit of normal, *VOD* veno-occlusive disease

### Monitoring and diagnosing veno-occlusive disease

The expert panel noted that the CIBMTR VOD risk stratification model does not include InO exposure, and all patients receiving InO should be considered to be at high risk of VOD. Therefore, these patients should be closely monitored for signs and symptoms of VOD to allow early diagnosis and treatment (Table 2). When patients develop mild VOD, they should be followed closely for progression because a patient’s severity classification could change rapidly [7]. Bilirubin levels should be monitored before each InO dose, and the patient’s weight should be measured, at a minimum, before each InO cycle. Liver function should also be monitored before and following each dose of InO. Close monitoring of these parameters should continue after transplant. Unexpected weight changes and/or changes in liver function should alert the physician to the possibility of VOD and should lead to further diagnostic workup before further treatment with InO. Additional VOD diagnostic techniques (e.g., abdominal ultrasound, measurement of the hepatic venous pressure gradient, transjugular liver biopsy [12] may be performed at the physician’s discretion. Diagnosing and monitoring VOD based on the EBMT VOD position statement is recommended [10].

### Managing veno-occlusive disease

In accordance with recommendations provided by the EBMT VOD position statement, supportive care to treat the symptoms of VOD is the first step in management [10]. Careful attention to fluid balance is recommended for all patients regardless of VOD severity. Symptomatic care with diuretics, oxygen, and hemodialysis/hemofiltration can also be used [10]. Paracentesis is recommended when ascites compromises respiration [10]. A transjugular intrahepatic portosystemic shunt can be considered in severe cases [10]. Patients should also continue to receive ursodiol prophylactically. Other than symptomatic treatments, defibrotide is the only agent approved in the United States and European Union for treatment of VOD with renal or pulmonary compromise [14, 15]. The recommended dose for defibrotide is 6.25 mg/kg every 6 h for a minimum of 21 days and should be continued until the signs and symptoms of VOD resolve (up to a maximum of 60 days) [14, 15]. Recommended InO dose modifications for non-hematologic toxicities should be followed (Table 3) [16].

**Table 3:** InO dose modifications for hematologic and non-hematologic toxicities [16]

Hematologic toxicities	
Criteria	Dose modification
If before InO treatment ANC was ≥1 × 10^9^/L	If ANC decreases, then interrupt the next cycle of treatment until recovery of ANC to ≥1 × 10^9^/L. Discontinue InO if low ANC persists for >28 days and is suspected to be related to InO.
If before InO treatment platelet count was ≥50 × 10^9^/L	If platelet count decreases, then interrupt the next cycle of treatment until platelet count recovers to ≥50 × 10^9^/L. Discontinue InO if low platelet count persists for >28 days and is suspected to be related to InO.
If before InO treatment ANC was <1 × 10^9^/L and/or platelet count was <50 × 10^9^/L^a^	If ANC or platelet count decreases, then interrupt the next cycle of treatment until at least 1 of the following occurs:
	• ANC and platelet count recover to at least baseline levels for the prior cycle, or
	• ANC recovers to ≥1 × 10^9^/L and platelet count recovers to ≥50 × 10^9^/L^a^, or
	• Stable or improved disease (based on most recent bone marrow assessment) and the ANC and platelet count decrease is considered to be due to the underlying disease (not considered to be InO-related toxicity)

Non-hematologic toxicities	
Toxicity	Dose modification
VOD or other severe liver toxicity	Permanently discontinue treatment
Total bilirubin >1.5 × ULN and AST/ALT >2.5 × ULN	Interrupt dosing until recovery of total bilirubin to ≤1.5 × ULN and AST/ALT to ≤ 2.5 × ULN before each dose unless due to Gilbert’s syndrome or hemolysis. Permanently discontinue treatment if total bilirubin dose not recover to ≤1.5 × ULN or AST/ALT does not recover to ≤ 2.5 × ULN
Infusion-related reaction	Interrupt the infusion and institute appropriate medical management. Depending on the severity of the infusion-related reaction, consider discontinuation of the infusion or administration of steroids and antihistamines. For severe or life-threatening infusion reactions, permanently discontinue treatment
Non-hematologic toxicity ≥Grade 2^b^	Interrupt treatment until recovery to Grade 1 or pre-treatment grade levels before each dose

*ALT* alanine aminotransferase, *AST* aspartate aminotransferase, *ANC* absolute neutrophil count, *InO* inotuzumab ozogamicin, *ULN* upper limit of normal, *VOD* veno-occlusive disease

^a^Platelet count used for dosing should be independent of blood transfusion

^b^Severity grade according to National Cancer Institute Common Terminology Criteria for Adverse Events, version 3.0

### Neutropenia and febrile neutropenia

Myelosuppressive systemic cancer chemotherapies commonly cause neutropenia and febrile neutropenia [17]. Neutropenia was the most common AE reported in the INO-VATE study (48% (any grade) with InO vs 44% (any grade) with standard therapy) [2]. Febrile neutropenia was also relatively common; any grade febrile neutropenia occurred in 27% of patients in the InO arm and 52% in the standard-therapy group [2].

### Recommendations

Complete blood counts should be performed before each InO dose, and patients should be monitored for signs and symptoms of neutropenia and febrile neutropenia (Table 4). Interruption, reduction, or discontinuation of InO doses may be necessary in patients with severe infection or severe neutropenia. Dose interruptions may also be required if a patient’s absolute neutrophil count (ANC) decreases with InO (Table 3). In patients with ANC < 1000/mm^3^, granulocyte colony-stimulating factor should be administered [18]. Prophylactic myeloid colony-stimulating factors may be considered for patients with an expected neutropenia or fever risk of ≥20% [19].

**Table 4:** Recommendations for monitoring and managing important adverse events associated with InO

*Neutropenia/febrile neutropenia*
• Monitor CBCs before each InO dose, monitor for signs and symptoms of infection or other effects of myelosuppression during treatment, and provide appropriate management
• Dose interruption, dose reduction, or permanent InO discontinuation may be required to manage severe infection or myelosuppression, including severe neutropenia (Table 3)
• InO dose interruption within a treatment cycle (i.e., day 8 and/or 15) due to neutropenia is not required
*Thrombocytopenia/bleeding events*
• Monitor CBCs before each InO dose and monitor for signs and symptoms of bleeding/hemorrhage or other effects of myelosuppression during treatment
• InO dose interruption within a treatment cycle (i.e., day 8 and/or 15) due to thrombocytopenia is not required
• Dose interruption, dose reduction, or permanent InO discontinuation may be required to manage bleeding/hemorrhage (Table 3)
*Infusion-related reactions*
• For severe or life-threatening infusion reactions, permanently discontinue InO
• If an infusion-related reaction occurs, interrupt the infusion and institute appropriate medical management
• Before InO dosing, premedication with a corticosteroid, antipyretic, and antihistamine is recommended
• Depending on the severity of the infusion-related reaction, consider discontinuing the infusion or administering steroids and antihistamines
*Tumor lysis syndrome*
• Monitor for signs and symptoms of tumor lysis syndrome and treat according to standard medical practice
• For patients with circulating lymphoblasts, cytoreduction with a combination of hydroxyurea, steroids, and/or vincristine to a peripheral blast count ≤10 000/mm^3^ is recommended before the first InO dose
*Prolonged QT syndrome*
• Administer InO with caution in patients with a history of or predisposition for prolonged QT syndrome, who are taking medicinal products known to prolong QT interval, or patients with electrolyte disturbances
• ECG results and electrolyte levels should be obtained before the start of InO treatment and periodically monitored during treatment
• Carefully consider and monitor the concomitant use of InO and medicinal products known to prolong QT interval or that are able to induce torsades de pointes

*ANC* absolute neutrophil count, *CBC* complete blood count, *ECG* electrocardiogram, *InO* inotuzumab ozogamicin

### Thrombocytopenia

Thrombocytopenia is a common adverse effect of cancer treatments [20]. Signs and symptoms of thrombocytopenia include easy bruising, melena, and rash [21]. On the basis of findings from the INO-VATE study, thrombocytopenia of any grade was reported in 45% of patients in the InO group compared with 61% of patients in the standard-therapy group [2]. Fewer patients in the InO group required platelet transfusions (64%) compared with the standard-therapy group (95%) [2]. In the InO group, hemorrhagic events were reported in 33% of patients [16].

### Recommendations

In patients receiving InO, monitoring of thrombocytopenia should be based on the aforementioned signs and symptoms and complete blood counts (Table 4). Moreover, InO dose interruptions, reductions, or discontinuations are recommended to manage low platelet counts and bleeding (Table 3).

### Infusion-related reactions

Infusion-related reactions can potentially occur in nearly all systemic cancer treatments and range in severity from mild flushing or rash to death [22]. On the basis of findings from the INO-VATE study, infusion-related reactions of any grade occurred in 1% of patients in the InO group and 2% of patients in the standard-therapy group [3]. Although the incidence of infusion-related reactions associated with InO is low, patients should be closely monitored during and immediately after an infusion to ensure prompt management if needed to reduce the risk of severe symptoms [22].

### Recommendations

Premedication with a corticosteroid, antipyretic, and antihistamine is recommended before InO administration (Table 4). Depending on the severity of the infusion-related reaction, interruption or discontinuation of the infusion should be considered. Treatment with InO should be discontinued in severe or life-threatening infusion reactions (Table 3) [16].

### Tumor lysis syndrome

Tumor lysis syndrome is more likely with the first dose of cancer treatment and is a potentially life-threatening emergency that can occur either spontaneously or because of cell death from cancer treatments [23, 24]. Tumor lysis syndrome of any grade was reported in 3% of patients in the InO arm compared with 2% of patients receiving standard therapy in the INO-VATE study [3]. Prompt detection and management of tumor lysis syndrome is important to prevent potentially fatal complications such as acute renal failure, arrhythmias, seizures, and death [23].

### Recommendations

Patients receiving InO should be monitored for signs and symptoms of tumor lysis syndrome and treated accordingly (Table 4). Cytoreduction before the first InO dose is recommended in patients with >10,000/mm^3^ circulating lymphoblasts. In patients who have baseline uric acid levels >7.5 mg/dl and in patients at high risk of developing tumor lysis syndrome, administration of rasburicase should be considered [23, 24]. Additional preventative measures (e.g., hydration, allopurinol) may also be considered if warranted [23].

### Prolonged QT syndrome

Torsades de pointes, associated with QT prolongation, is a potentially sudden and fatal arrhythmia [25]. Some common medications known to cause QT prolongation include antiarrhythmic drugs (e.g., quinidine, procainamide, amiodarone), antibiotics (e.g., macrolides, ketoconazole), antihistamines (e.g., terfenadine, astemizole), antidepressants (e.g., tricyclic antidepressants), and antipsychotics (e.g., haloperidol) [25]. In the INO-VATE trial, prolonged QT syndrome of any grade was reported in 2% of patients in the InO group and none of the patients in the standard-therapy group [3]. Although QT prolongation is not commonly associated with InO, it is important to monitor for and attempt to prevent this potentially life-threatening syndrome.

### Recommendations

Electrolyte levels and electrocardiogram results should be monitored before starting InO and periodically throughout treatment (Table 4). InO should be used with caution in patients with electrolyte imbalances or a history of prolonged QT syndrome and in recipients of medications known to prolong the QT interval.

## Conclusions

The INO-VATE study reported clinically important AEs associated with InO [2]. VOD is the AE of greatest concern because it was substantially more common with InO than with standard salvage chemotherapy in the INO-VATE study, whereas the incidences of other AEs associated with InO were lower or similar to standard therapy.

An expert panel of oncologists and hematologists concluded that patients receiving InO who proceed to follow-up HCT are at a high risk of developing VOD, up to ~20%, and should be monitored and managed accordingly. To prevent VOD, prophylactic pharmacologic agents are recommended, and patients for whom HCT is anticipated should limit their number of InO cycles to 2 if feasible. In addition, in patients who do develop VOD, management should be provided in accordance with the EBMT VOD position statement recommendations [10].

Neutropenia, febrile neutropenia, and thrombocytopenia are commonly reported with InO therapy. Consequently, physicians should monitor the patient’s complete blood count before each InO dose and monitor for signs and symptoms of myelosuppression or bleeding throughout InO treatment. Dosing adjustments or permanent discontinuations of InO are recommended if necessary to manage these AEs.

Although infusion-related reactions, tumor lysis syndrome, and QT prolongation are not commonly reported in patients receiving InO, they are serious and potentially life-threatening conditions that physicians need to closely monitor to ensure that early management is provided. Before InO dosing, premedication may help prevent infusion-related reactions, and cytoreduction can prevent tumor lysis syndrome. In addition, InO should be used with caution in patients with a history of QT prolongation, electrolyte imbalances, and in patients who are taking concomitant medications that prolong the QT interval.

Overall, the risk of these clinically important AEs associated with InO can be mitigated with preventative measures and prompt diagnosis and management.

## Electronic supplementary material

Supplemental Table 1
