# Supplementary material for: Management of important adverse events associated with inotuzumab ozogamicin: expert panel review
Source: Bone Marrow Transplant. 2018 Jan 12;53(4):449–56. doi: 10.1038/s41409-017-0019-y (PMC5897380; doi:10.1038/s41409-017-0019-y)
Supplement: Supplementary file 1 — Supplemental Table 1 [file 41409_2017_19_MOESM1_ESM.docx]

**Supplemental Table 1. All Cause and Treatment-Related Treatment-Emergent Adverse Events from the INO-VATE Trial** (2)

|  | **InO (n=139)** | | | | **Standard Therapy (n=120)** | | | |
| --- | --- | --- | --- | --- | --- | --- | --- | --- |
|  | **All-Cause** | | **Treatment-Related** | | **All-Cause** | | **Treatment-Related** | |
|  | **All Grade** | **Grade ≥3** | **All Grade** | **Grade ≥3** | **All Grade** | **Grade ≥3** | **All Grade** | **Grade ≥3** |
| Any AE,*^†^ n (%) | 136 (98) | 126 (91) | 119 (86) | 96 (69) | 119 (99) | 114 (95) | 109 (91) | 93 (78) |
| Thrombocytopenia | 62 (45) | 51 (37) | 40 (29) | 28 (20) | 73 (61) | 71 (59) | 57 (48) | 56 (47) |
| Neutropenia | 67 (48) | 64 (46) | 50 (36) | 47 (34) | 53 (44) | 50 (42) | 46 (38) | 43 (36) |
| Anemia | 42 (30) | 26 (19) | 25 (18) | 15 (11) | 64 (53) | 48 (40) | 46 (38) | 35 (29) |
| Nausea | 44 (32) | 3 (2) | 21 (15) | 0 | 56 (47) | 0 | 41 (34) | 0 |
| Febrile neutropenia | 37 (27) | 33 (24) | 22 (16) | 20 (14) | 62 (52) | 59 (49) | 51 (43) | 48 (40) |
| Pyrexia | 37 (27) | 5 (4) | 15 (11) | 2 (1) | 51 (43) | 6 (5) | 30 (25) | 3 (3) |
| Leukopenia | 38 (27) | 35 (25) | 23 (17) | 21 (15) | 47 (39) | 47 (39) | 31 (26) | 31 (26) |
| Diarrhea | 25 (18) | 1 (1) | 8 (6) | 0 | 48 (40) | 1 (1) | 27 (23) | 1 (1) |
| Headache | 39 (28) | 2 (1) | 13 (9) | 1 (1) | 33 (28) | 0 | 9 (8) | 0 |
| Lymphopenia | 24 (17) | 22 (16) | 15 (11) | 15 (11) | 34 (28) | 34 (28) | 22 (18) | 22 (18) |
| Vomiting | 24 (17) | 1 (1) | 10 (7) | 0 | 28 (23) | 0 | 19 (16) | 0 |
| Constipation | 23 (17) | 0 | 9 (7) | 0 | 28 (33) | 0 | 9 (8) | 0 |
| Fatigue | 31 (22) | 4 (3) | 13 (9) | 2 (1) | 17 (14) | 2 (2) | 12 (10) | 1 (1) |
| Hypokalemia | 23 (17) | 10 (7) | 7 (5) | 3 (2) | 23 (19) | 3 (3) | 11 (9) | 2 (2) |
| AST increased | 28 (20) | 7 (5) | 13 (9) | 1 (1) | 12 (10) | 4 (3) | 5 (4) | 1 (1) |
| Insomnia | 21 (15) | 0 | 6 (4) | 0 | 18 (15) | 0 | 2 (2) | 0 |
| Abdominal pain | 19 (14) | 3 (2) | 5 (4) | 1 (1) | 20 (17) | 1 (1) | 11 (9) | 1 (1) |
| Rash | 13 (9) | 0 | 4 (3) | 0 | 23 (19) | 0 | 13 (11) | 0 |
| Cough | 15 (11) | 0 | 0 | 0 | 21 (18) | 1 (1) | 4 (3) | 0 |
| GGT increased | 24 (17) | 12 (9) | 16 (12) | 6 (4) | 9 (8) | 5 (4) | 1 (1) | 1 (1) |
| Hyperbilirubinemia | 21 (15) | 5 (4) | 10 (7) | 4 (3) | 12 (10) | 4 (3) | 6 (5) | 3 (3) |
| Epistaxis | 21 (15) | 1 (1) | 5 (4) | 1 (1) | 11 (9) | 2 (2) | 3 (3) | 0 |
| ALT increased | 19 (14) | 4 (3) | 10 (7) | 1 (1) | 13 (11) | 4 (3) | 5 (4) | 0 |
| Hypotension | 11 (8) | 0 | 3 (2) | 0 | 20 (17) | 5 (4) | 4 (3) | 1 (1) |
| Appetite decreased | 13 (9) | 2 (1) | 6 (4) | 2 (1) | 16 (13) | 3 (3) | 12 (10) | 2 (2) |
| Chills | 14 (10) | 0 | 6 (4) | 0 | 14 (12) | 0 | 8 (7) | 0 |
| Pain in extremity | 12 (9) | 0 | 0 | 0 | 15 (13) | 1 (1) | 4 (3) | 1 (1) |
| Dizziness | 12 (9) | 0 | 3 (2) | 0 | 13 (11) | 0 | 4 (3) | 0 |
| Asthenia | 13 (9) | 3 (2) | 5 (4) | 2 (1) | 12 (10) | 2 (2) | 5 (4) | 0 |
| Peripheral edema | 12 (9) | 1 (1) | 1 (1) | 0 | 12 (10) | 0 | 3 (3) | 0 |
| Dyspnea | 7 (5) | 1 (1) | 1 (1) | 0 | 16 (13) | 2 (2) | 4 (3) | 0 |
| ALP increased | 16 (12) | 2 (1) | 8 (6) | 1 (1) | 7 (6) | 1 (1) | 4 (3) | 1 (1) |
| Hypocalcemia | 11 (8) | 2 (1) | 2 (1) | 1 (1) | 12 (10) | 3 (3) | 3 (3) | 1 (1) |
| Mucosal inflammation | 5 (4) | 1 (1) | 3 (2) | 1 (1) | 15 (13) | 3 (3) | 11 (9) | 2 (2) |
| Tachycardia | 6 (4) | 0 | 1 (1) | 0 | 12 (10) | 1 (1) | 2 (2) | 0 |
| VOD | 15 (11) | 13 (9) | 13 (9) | 11 (8) | 1 (1) | 1 (1) | 0 | 0 |
| Lipase increased | 14 (10) | 6 (4) | 10 (7) | 4 (3) | 0 | 0 | 0 | 0 |

AE=adverse event; ALP=alkaline phosphatase; ALT=alanine aminotransferase; AST=aspartate aminotransferase; GGT=gamma-glutamyltransferase; InO=inotuzumab ozogamicin; VOD=veno-occlusive disease.

*Data represent the safety population (data cutoff date of October 2, 2014); adverse events were graded according to the National Cancer Institute Common Terminology Criteria for Adverse Events version 3.0.

^†^All-cause AEs with ≥10% incidence occurring in either arm in the safety population (any treatment cycle) in descending order of total frequency across arms.
